# A review on personal calibration issues for video-oculographic-based gaze tracking

**DOI:** 10.3389/fpsyg.2024.1309047

**Published:** 2024-03-20

**Authors:** Jiahui Liu, Jiannan Chi, Zuoyun Yang

**Affiliations:** ^1^School of Automation and Electrical Engineering, University of Science and Technology Beijing, Beijing, China; ^2^Beijing Engineering Research Center of Industrial Spectrum Imaging, University of Science and Technology Beijing, Beijing, China; ^3^Shunde Innovation School, University of Science and Technology Beijing, Foshan, China

**Keywords:** personal calibration, video-oculographic, gaze tracking, eye-movement interaction, visual information

## Abstract

Personal calibration is a process of obtaining personal gaze-related information by focusing on some calibration benchmarks when the user initially uses a gaze tracking system. It not only provides conditions for gaze estimation, but also improves gaze tracking performance. Existing eye-tracking products often require users to conduct explicit personal calibration first, thereby tracking and interacting based on their gaze. This calibration mode has certain limitations, and there is still a significant gap between theoretical personal calibration methods and their practicality. Therefore, this paper reviews the issues of personal calibration for video-oculographic-based gaze tracking. The personal calibration information in typical gaze tracking methods is first summarized, and then some main settings in existing personal calibration processes are analyzed. Several personal calibration modes are discussed and compared subsequently. The performance of typical personal calibration methods for 2D and 3D gaze tracking is quantitatively compared through simulation experiments, highlighting the characteristics of different personal calibration settings. On this basis, we discuss several key issues in designing personal calibration. To the best of our knowledge, this is the first review on personal calibration issues for video-oculographic-based gaze tracking. It aims to provide a comprehensive overview of the research status of personal calibration, explore its main directions for further studies, and provide guidance for seeking personal calibration modes that conform to natural human-computer interaction and promoting the widespread application of eye-movement interaction.

## 1 Introduction

With the rapid development of artificial intelligence, eye-movement interaction is increasingly favored by people due to its efficient and real-time. Eye-movement interaction utilizes gaze information for interaction and its key technology is gaze tracking, which uses visual information from face or eye images to analyze the user's gaze direction or point-of-regard (POR). At present, gaze tracking has been used in human-computer interaction, medical diagnosis, virtual reality, and intelligent transportation (Drakopoulos et al., 2021; Hu et al., 2022; Li et al., 2022).

**Figure 1:**
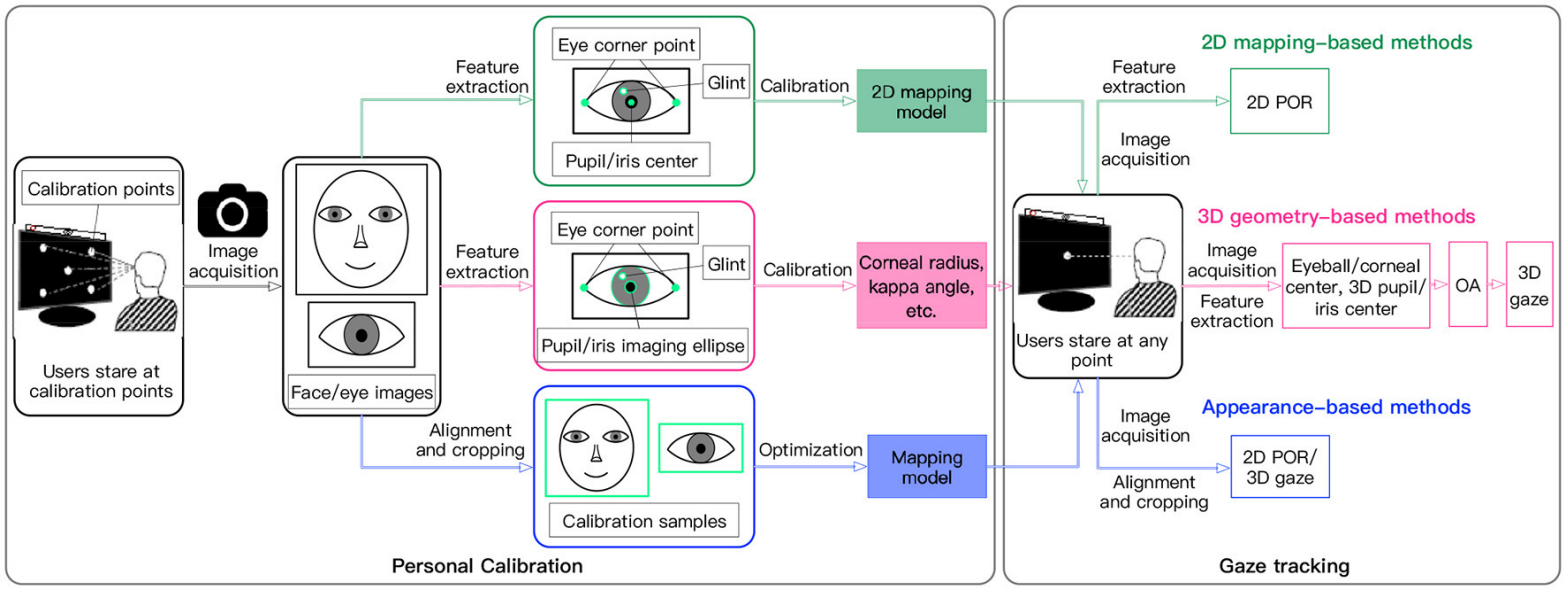
Personal calibration processes in different methods.

Video-oculographic (VOG)-based gaze tracking systems generally conduct a personal calibration process to obtain some user-specific parameters or images, as shown in Figure 1. 2D mapping-based methods take the eye invariant features (e.g., eye corner point or glint) as the benchmark, and construct a mapping model between eye variation features (e.g., pupil center or iris center) and 2D POR for gaze estimation. To determine the mapping model for a specific user, it is necessary to calibrate some of his/her specific parameters through personal calibration. At present, most personal calibration methods require users to stare at multiple calibration points on the screen, so as to obtain sufficient calibration data for mapping model construction (Cheng et al., 2017; Mestre et al., 2018; Hu et al., 2019; Uhm et al., 2020). Hu et al. (2019) asked users to focus on nine explicit calibration points while keeping their heads fixed during personal calibration, to estimate the POR using the eye-movement vector between iris center and corner points. Uhm et al. (2020) used four calibration points to calibrate the projection transformation matrix between eye image and screen.

3D geometry-based methods utilize visual features (such as pupil, iris, and glints) to calibrate eye invariant parameters (such as corneal radius and kappa angle) based on eyeball structure and geometric imaging model. Then, eye-variation parameters (such as eyeball center, corneal center, pupil center, and iris center) are estimated to reconstruct the optical axis (OA) of the eyeball, thereby determining the visual axis (VA) using the OA and the kappa angle. Due to a fixed deviation between the OA and the VA, called the kappa angle, at least a single-point calibration is generally required to calculate it (Lai et al., 2015). To estimate the VA in a simple system, some parameters such as corneal radius, distance between corneal center and pupil center also need to be calibrated, in addition to the kappa angle (Cristina and Camilleri, 2016; Zhou et al., 2017). Cristina and Camilleri (2016) detected a frontal eye and head pose during personal calibration, and then estimated the 3D gaze from a single camera based on a cylindrical head and spherical eyeball model.

Appearance-based methods take face or eye images as input, and learn the mapping of face or eye images to gaze information by using a large number of training samples with ground-truth labels, thereby predicting the gaze information for new images using the trained model. Most methods are calibration-free, but some methods use a few calibration samples to optimize the model and reduce the impact of individual differences (Krafka et al., 2016; Gu et al., 2021; Liu G. et al., 2021; Wang et al., 2023). Liu G. et al. (2021) trained a differential convolutional neural network to predict the gaze difference between two input eye images of a same subject, and the gaze direction of a new eye sample was predicted by inferring the gaze differences of a set of subject-specific calibration images. Krafka et al. (2016) used the data from 13 fixed locations for calibration to train the SVR to predict the gaze location. The performance was improved significantly.

Overall, the roles of personal calibration are mainly reflected in three aspects: (1) calibrate some user-specific parameters in the gaze estimation model to meet the condition for gaze estimation; (2) calibrate the user-specific parameters using multiple calibration benchmarks to enhance its robustness; (3) introduce some user-specific features to improve the gaze accuracy. At present, eye tracking products on the market also require personal calibration at the beginning of use, by looking at several calibration points or images. However, this explicit user calibration limits the convenience of product use and provides a poor user experience. To help improve the personal calibration process for gaze tracking systems and promote the implementation of instant use that an ideal gaze tracking system should have, we specifically explore the issues of personal calibration for gaze tracking. This paper provides a comprehensive overview of calibration information, calibration settings, and calibration modes involved in personal calibration, clarifies the characteristics of typical personal calibration methods under different calibration settings through simulation experiments, and discusses some key issues for designing personal calibration. In view of the research status, some main directions for future studies on personal calibration are provided. To the best of our knowledge, there is no literature specifically exploring the issues of personal calibration for gaze tracking. Most literature only provides a short description of its own calibration process. The main contributions of this work are as follows:

(1) This is the first review on the issues of personal calibration for gaze tracking. Some calibration information, calibration settings, and calibration modes in existing methods are summarized, reflecting the characteristics of current personal calibration.
(2) Simulation experiments of several typical personal calibration methods are conducted to clarify the key issues of personal calibration, which are helpful in determining the calibration information, setting the calibration process, and selecting the calibration mode.
(3) The current limitations and further research directions of personal calibration are discussed, providing guidance for researchers to seek more convenient and natural personal calibration methods, and promoting the upgrading of personal calibration in eye-movement interaction applications.

**Figure 2:**
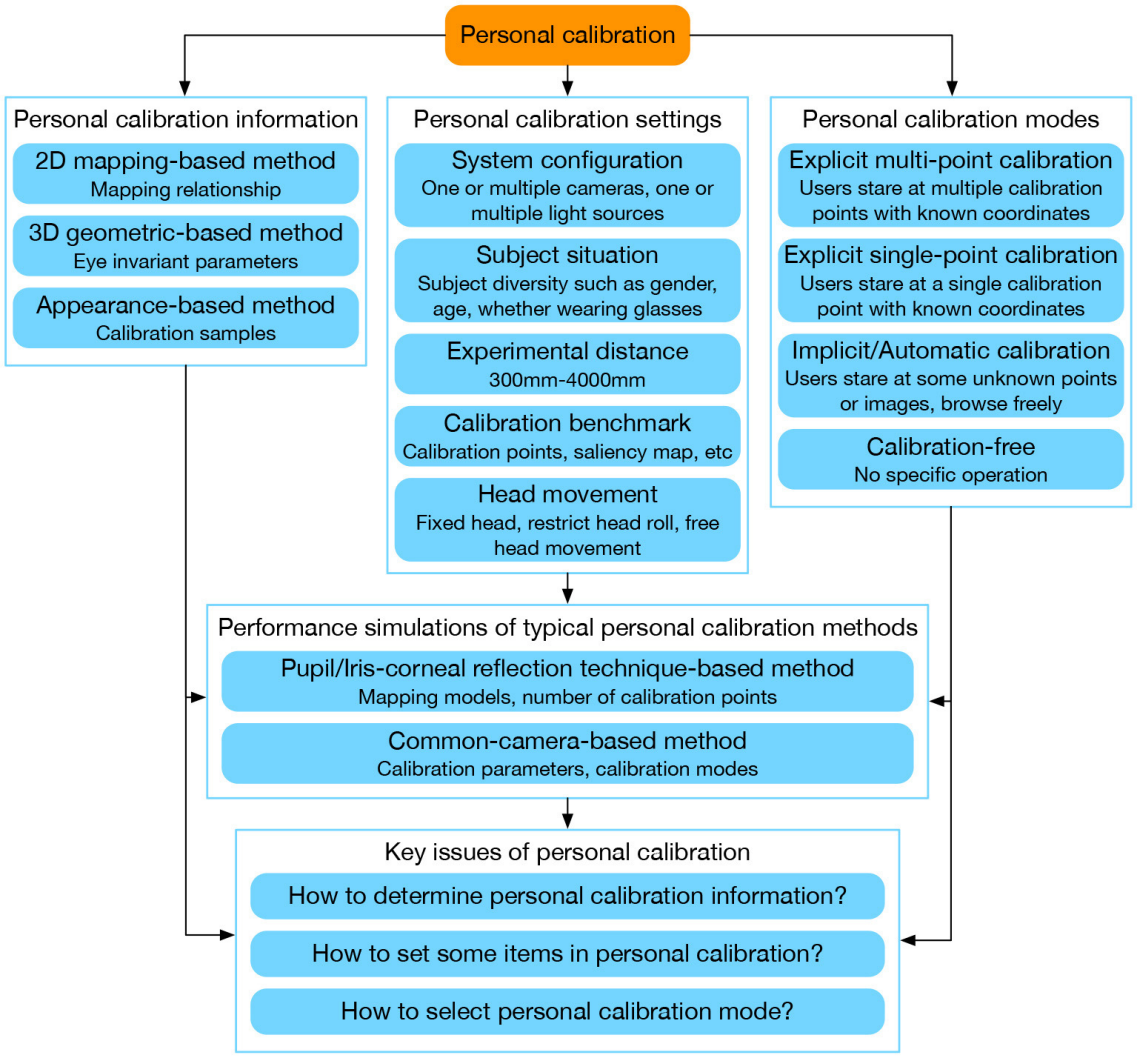
Framework of this paper.

This paper is organized as shown in Figure 2. Section 2 provides an overview of the user-specific information obtained through personal calibration in different methods. Section 3 analyzes some of the main settings in the existing personal calibration process. We summarize several existing personal calibration modes for gaze tracking and compared their characteristics in Section 4. Section 5 compares the performance of typical personal calibration methods under different settings through simulation experiments, reflecting the characteristics of different personal calibration settings intuitively. A discussion of the key issues for designing personal calibration is given in Section 6. Finally, this paper is concluded and the development trends of personal calibration are prospected in the conclusion.

## 2 Personal calibration information

**Figure 3:**
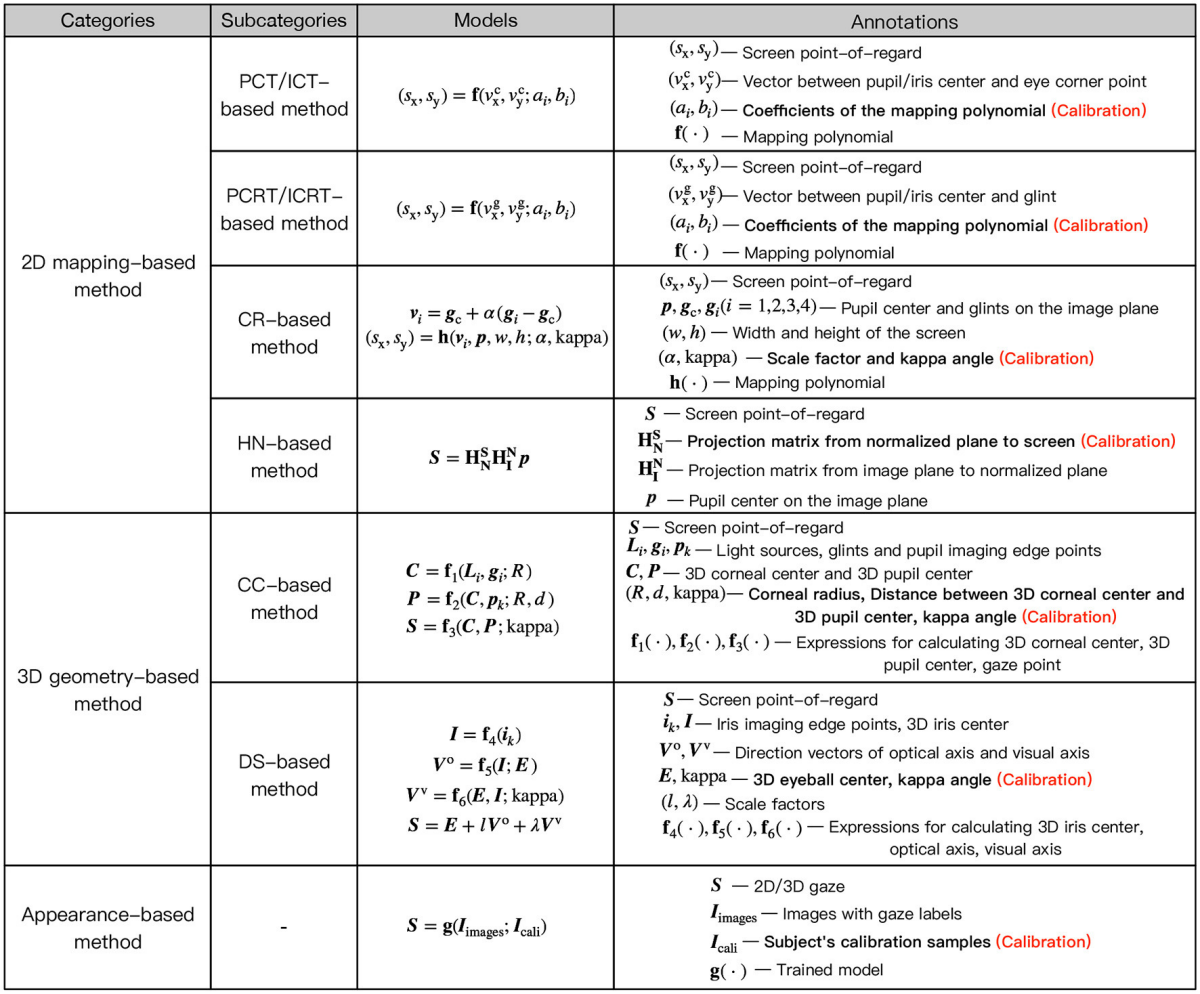
Personal calibration information in different methods.

The primary role of personal calibration is to provide useful personal information for gaze estimation. According to the classification of gaze estimation methods, this section discusses the commonly calibrated personal information in different methods, as Figure 3 shows.

### 2.1 Calibration information in 2D mapping-based methods

2D mapping-based methods mainly include pupil/iris-corner technique (PCT/ICT)-based methods, pupil/iris-corneal reflection technique (PCRT/ICRT)-based methods, cross-ratio (CR)-based methods, and homography normalization (HN)-based methods.

PCT/ICT-based methods construct a 2D mapping model between the vector formed by pupil/iris center and eye corner point and the POR. To determine the mapping model, the users need to sequentially stare at multiple on-screen calibration points during personal calibration to collect multiple sets of corresponding pupil/iris-corner vector and POR, so as to calibrate the user-specific coefficients of mapping model through regression (Cheung and Peng, 2015; George and Routray, 2016; Hu et al., 2019). PCRT/ICRT-based methods utilize the glint as the reference point, and construct a 2D mapping model between the vector formed by pupil/iris center and glint and the POR (Sigut and Sidha, 2011; Blignaut, 2013; Rattarom et al., 2017; Mestre et al., 2018). Similar to PCT/ICT-based methods, after determining the coefficients of mapping model through personal calibration, the POR can be estimated by substituting the pupil/iris-glint vector into the determined mapping model. The complexity of personal calibration for polynomial mapping methods such as PCT/ICT-based methods and PCRT/ICRT-based methods is related to the mapping model used. The most commonly used mapping model is the quadratic polynomial of the two components of the eye movement feature vector. There are 12 coefficients in this quadratic polynomial model, which uses nine calibration points for personal calibration. Cerrolaza et al. (2008) analyzed more than 400,000 mapping models, and demonstrated that higher-order polynomials cannot significantly improve the performance. A simple mapping model can still obtain ideal gaze accuracy (Jen et al., 2016; Xia et al., 2016). The simpler the polynomial, the fewer coefficients that need to be calibrated, and the fewer calibration points required for personal calibration. Xia et al. (2016) used a simple linear mapping between the pupil center and the screen point, and determined the mapping model using two calibration points.

CR-based methods use the invariance property of cross-ratios in projective transformations. The corneal reflection plane formed by the corneal reflections of light sources attached to four corners of the screen is taken as the medium, and the on-screen point corresponding to the pupil imaging center is calculated due to the equal CR of corresponding edges on the image plane and the screen, which is regarded as the gaze point (Coutinho and Morimoto, 2013; Arar et al., 2015, 2017; Cheng et al., 2017). Since the fact that the 3D pupil center and the corneal reflection points are not coplanar, it is necessary to determine a scale factor α through personal calibration to make the pupil center coplanar to the corneal reflection plane (Coutinho and Morimoto, 2013). In addition, the mapping point of the pupil imaging center on the screen is regarded as the gaze point, which is essentially the intersection point of the OA and the screen (POA), rather than the actual POR defined as the intersection of the VA and the screen. Therefore, it is also common to calibrate the kappa angle between the OA and the VA of the eyeball. Arar et al. (2015) introduced a personal calibration method using regularized least squares regression to compensate the kappa angle after using the conventional CR-based method to estimate the initial gaze point.

HN-based methods utilize two homography projection transformations from the image plane to the corneal reflection plane, as well as from the corneal reflection plane to the screen, to convert the pupil imaging center to a point on the screen, which is the gaze point. Due to the uncalibrated system, the corneal reflection plane is unknown, so it is necessary to define a normalized plane to replace it. The homography matrix from the image plane to the normalized plane is calculated by four glints generated by corneal reflection and four corner points of normalized plane. And the homography matrix from the normalized plane to the screen is determined through personal calibration using not less than four calibration points (Hansen et al., 2010; Shin et al., 2015; Morimoto et al., 2020).

### 2.2 Calibration information in 3D geometry-based methods

According to the type of camera used, 3D geometry-based methods can be divided into common-camera (CC)-based methods and depth-sensor (DS)-based methods.

Among the CC-based methods, the most common one is based on corneal reflection and pupil refraction (CRPR-based method). When a single-camera system is used, it is necessary to calibrate the corneal radius, the distance between 3D corneal center and 3D pupil center, and the kappa angle. In this way, the 3D corneal center and 3D pupil center can be estimated using the calibrated corneal radius and distance between 3D corneal center and 3D pupil center. The OA of the eyeball is represented by the line connecting 3D corneal center and 3D pupil center. Then the VA of the eyeball, that is the 3D gaze, can be converted from the OA using the calibrated kappa angle (Guestrin and Eizenman, 2006; Brousseau et al., 2018; O'Reilly et al., 2019). When a multi-camera system is used, 3D corneal center estimation can be simplified as the intersection of the reflection planes of two light sources of two cameras, and the OA of the eyeball can be obtained by the intersection of the refraction planes composed of 3D corneal center, camera optical center, and corresponding pupil imaging center of two cameras. Therefore, only the kappa angle is necessary to be calibrated to estimate the 3D gaze (Villanueva and Cabeza, 2008; Lidegaard et al., 2014). The personal calibration process of this method can be simplified to single-point calibration by fully utilizing the eyeball structure and the geometric imaging model (Villanueva and Cabeza, 2008; Lai et al., 2015). In addition, since the iris radius is an eye invariant parameter and the iris is less affected by corneal refraction (Hansen and Ji, 2010), 3D gaze estimation can be achieved using a single-camera system by calibrating the iris radius, corneal radius, and the kappa angle (Liu J. et al., 2020). In the absence of active light source, Cristina and Camilleri (2016) estimated the 3D gaze using a single camera based on a cylindrical head and spherical eye model, where the personal calibration information was detecting an initial frontal eye and head pose.

DS-based methods usually only require a depth camera such as Kinect. Due to the ability to obtain depth information, this method can estimate the head pose to obtain the transformation matrix between head and camera coordinate systems. According to the property that the eyeball center remains fixed relative to the head, the eyeball center in the head coordinate system can be calculated through personal calibration, and the 3D eyeball center in the camera coordinate system can be transformed. Combined with the 3D pupil or iris center calculated using the geometric imaging model, 3D corneal center can be determined and the OA of the eyeball can be constructed. By adding the kappa angle obtained from personal calibration, the VA of the eyeball can be calculated. The 3D POR can be obtained by intersecting the VA and the screen (Sun et al., 2014, 2015; Wang and Ji, 2016; Zhou et al., 2017). To fully consider individual differences, in addition to calibrating the eyeball center in the head coordinate system and the kappa angle, Sun et al. (2015, 2014) calibrated the eyeball radius, and the vector from eyeball center to inner eye corner in the head coordinate system. Wang and Ji (2016) additionally calibrated the eyeball radius and the distance between eyeball center and corneal center, where the distance between eyeball center and corneal center was used to estimate the 3D corneal center to represent the real gaze.

### 2.3 Calibration information in appearance-based methods

Appearance-based methods construct a mapping model between facial or eye appearance and the gaze. With the increase of the datasets available online, researchers can directly use existing datasets to conduct research on appearance-based gaze estimation. Due to the use of a large number of labeled training samples, this method usually does not require personal calibration to train a gaze estimation model. However, due to differences in eye appearance, illumination conditions, head pose, and distributions, this method requires sufficiently large and diverse data to produce accurate results (Uhm et al., 2020). Sugano et al. (2014) compared the mean estimation errors of random forest regression with person-specific training and cross-person training, which were 3.9° and 6.5°, respectively. This reflects the significant impact of individual differences and the limitations of the trained gaze estimation model. Therefore, some researchers used some calibration samples to train user-specific gaze estimation models (Zhang et al., 2015, 2019). In addition, some researchers used a few calibration samples for model calibration to improve the gaze estimation performance (Krafka et al., 2016; Park et al., 2018; He et al., 2019; Lindén et al., 2019; Chen and Shi, 2020; Gu et al., 2021; Liu G. et al., 2021; Wang et al., 2023). Lindén et al. (2019) modeled personal variations as a low-dimensional latent parameter space, and captured the range of personal variations by calibrating a spatial adaptive gaze estimator for a new person. Chen and Shi (2020) proposed to decompose the gaze angle into a subject-dependent bias term and a subject-independent gaze angle. During the test, the subject-dependent bias was estimated using a few images of the subject staring at a certain point, and then the gaze was represented by adding it to the calculated gaze angle. To alleviate the problem of information loss in the low-resolution gaze estimation task, Zhu et al. (2023) used the relatively fixed structure and components of human faces as prior knowledge and constructed a residual branch to recover the residual information between the low- and high-resolution images.

### 2.4 Discussion

Personal calibration is included in 2D mapping-based methods, 3D geometry-based methods, and appearance-based methods. The personal calibration process in appearance-based methods is the simplest, as it only requires the collection of calibration samples through the user's staring process, without the need for fine segmentation of image features. 2D mapping-based methods and 3D geometry-based methods need to detect visible features from the image and extract visible feature parameters, so as to calibrate specific parameters. 2D mapping-based methods use some feature center coordinates or feature points, such as the pupil center and glint, while 3D geometry-based methods often use some edge information of visual features on this basis, such as pupil imaging ellipse or pupil edge points. Therefore, comparatively, the personal calibration process in 3D geometry-based methods has the highest requirements on image processing, followed by 2D mapping-based methods. The personal calibration process in appearance-based methods has the lowest requirements.

From a performance perspective, compared to appearance-based methods, the most significant advantage of 2D mapping-based methods and 3D geometry-based methods is their high accuracy. Although both the 2D mapping-based method and the appearance-based method are mapping models, the 2D mapping-based method studies the essence of eye movement and determines the specific relationship between eye movement changes and gaze point through personal calibration. 3D geometry-based methods fully utilize visual features that reflect individual differences and eye movement changes. The calibration information mainly consists of some eye invariant parameters, which are relatively stable and not affected by head movement. It can be better adapted to the influence of head movement than 2D mapping-based methods that calibrate some mapping coefficients.

## 3 Personal calibration settings

**Figure 4:**
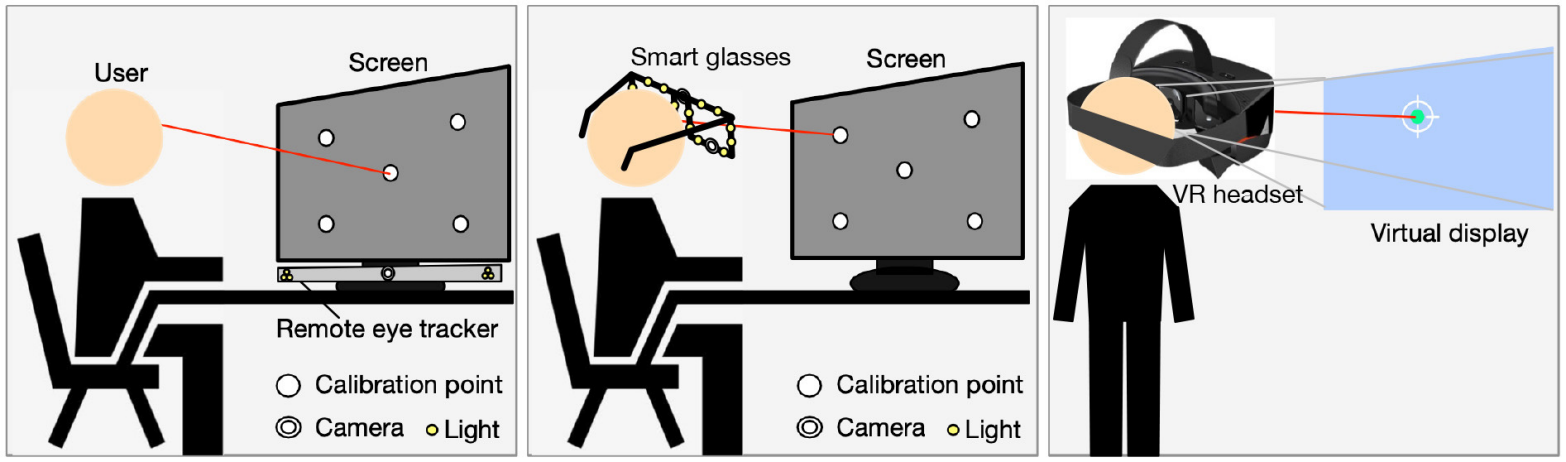
Schematic diagram of personal calibration.

Personal calibration is a process that is conducted before gaze tracking. Figure 4 shows the schematic diagram of personal calibration when users use different gaze tracking systems. When using a remote system (e.g., a laptop or a desktop eye-tracker combined with an external screen), the system camera, light sources, and screen are in close distance or nearly coplanar. The user needs to sit at a certain distance from the system and stare at some calibration points. During the user's staring process, the system will synchronously collect the user's face images, from which the user's facial or eye features can be obtained. The remote system has low interference to the user. When using smart glasses or a near-eye gaze tracker with a chin rest, the camera's optical axis deviates significantly from the gaze direction. The user is asked to stare at some calibration points a certain distance away while wearing the smart glasses or putting the head on the chin rest. During the staring process, the images of each eye are captured by a corresponding camera, and the features including pupil and glints can be extracted. When using a VR helmet, the helmet is equipped with a built-in display, and users can select the calibration function using the handle. The calibration process usually involves the user staring at several green dots that appear in sequence on the display, which is less affected by head movement. Alternatively, the user is asked to look at some displayed images (Chen and Ji, 2015; Wang et al., 2016; Alnajar et al., 2017; Hiroe et al., 2018). Personal calibration settings mainly involve system configuration, subject situation, experimental distance, calibration benchmark, and head movement. Table 1 lists the personal calibration settings mentioned in some typical methods, where “NA” represents those that are not mentioned or not available. In summary, the characteristics of personal calibration settings are reflected in:

**Table 1:** Personal calibration settings in some typical methods.

**Literature**	**Methods**	**System configuration**	**Subject situation**	**Experimental distance/mm**	**Calibration benchmark**	**Head movement/mm**
Hu et al. (2019)	PCT/ICT-based	1 camera	NA	600	9 calibration points	Fixed
Xia et al. (2016)	PCT/ICT-based	1 camera	10 subjects, wearing glasses is not allowed	860	12 calibration points (can be calibrated using 2 points)	Small
Blignaut (2013)	PCRT/ICRT-based	1 camera, 1 light source	26 subjects	800	5/9/14/18/23/135 calibration points	Fixed
Mestre et al. (2018)	PCRT/ICRT-based	2 cameras, 24 infrared LEDs	20 subjects, 31.9 ± 9.5 years old, with normal or corrected-to-normal visual acuity	NA	9 calibration points	Fixed
Cheng et al. (2017)	CR-based	1 camera, 5 light sources	6 subjects	600	9 calibration points	200 × 100 × 200
Uhm et al. (2020)	CR-based	1 camera, 4 light sources	8 subjects, 20–30 years old, with normal or corrected-to-visual acuity	600	4 calibration points	Free
Ma et al. (2014)	HN-based	1 camera, 4 light sources	6 subjects (3 males and 3 females), 25–35 years old, 3 of them make daily use of corrective lenses	700	9 calibration points	Fixed
Shin et al. (2015)	HN-based	1 camera, 1 light source	6 subjects	600	9 calibration points	Fixed/Free
Liu J. et al. (2021)	CC-based	1 camera, 1 light source	7 subjects, wearing glasses is not allowed	350–600	2 calibration points	Head roll is not allowed
Wang and Ji (2018)	CC-based	1 camera, 4 light sources	8 subjects (5 males and 3 females), 22–30 years old	500	Four theoretical constraints	Horizontal and vertical
O'Reilly et al. (2019)	CC-based	1 camera, 9 light sources	6 subjects	530	9 calibration points	Static calibration: fixed; natural calibration: 110 × 90 × 60
Lai et al. (2015)	CC-based	2 cameras, 2 light sources	6 subjects without glasses	500	1 calibration point	50 × 25 × 50
Wang and Ji (2016)	DS-based	Kinect	6 subjects without glasses	800	5 calibration points	Free
Sun et al. (2015)	DS-based	Kinect	8 subjects	550	1 calibration point	300 × 300 × 200
Li and Li (2016)	DS-based	Kinect	10 subjects	600	RGB camera while head in different positions	Horizontal and vertical, rotation is not allowed
Chen and Shi (2023)	Appearance-based	1 camera	21 subjects (11 males and 10 females), 10 of them wear glasses	900	1/5/9/16/32/64/128 calibration points	Free
Chen and Ji (2015)	Appearance-based	1 camera, 2 light sources	10 subjects	450–700	Saliency map	Free
Liu M. et al. (2020)	Appearance-based	(helmet) 3 cameras, 2 light sources	10 subjects	1,000–4,000	Saliency map	Free

### 3.1 System configuration

The system configuration used for personal calibration is consistent with the studied gaze tracking system, and is usually determined by the gaze tracking algorithm. Except for some scenarios that require high gaze accuracy using multi-camera systems, most current methods use single-camera systems as the researchers continue to explore the simplest system configuration for gaze tracking. To ensure sufficient conditions for personal calibration and gaze estimation, many methods require the use of a single-camera-multi-light-source system. The methods that can estimate gaze by using a single-camera system include PCT/ICT-based methods (Cheung and Peng, 2015; Xia et al., 2016; Eom et al., 2019; Hu et al., 2019), DS-based methods (Sun et al., 2014, 2015; Xiong et al., 2014; Li and Li, 2016; Wang and Ji, 2016; Zhou et al., 2016, 2017), and appearance-based methods (Alnajar et al., 2017; Kellnhofer et al., 2019; Zhang et al., 2019; Chen and Shi, 2023). For example, Hu et al. (2019) used a single camera to construct a polynomial mapping function from the eye-movement vector to the gaze point, where the eye-movement vector was represented by the average of four vectors formed by the inner and outer corners of both eyes and the iris center. The coefficients of mapping function were determined through nine-point calibration, and the obtained function was used for gaze estimation. Sun et al. (2014) used a Kinect to calibrated the eyeball radius and the vector from eyeball center to eye inner corner in the head coordinate system online, thereby estimating the eyeball center and the iris center in the camera coordinate system to indicate the gaze. They also calibrated the kappa angle to consider the individual differences (Sun et al., 2015). Zhang et al. (2019) proposed the GazeNet architecture to train a mapping model from 2D head angle and eye image to the gaze angles. The performance of cross-dataset evaluation improved by 22% on MPIIGaze, whose data was collected during laptop use.

### 3.2 Subject situation

To verify the generality of the algorithm, individual differences among subjects should be fully considered, such as race, gender, age, memory, and experience. At present, most methods do not consider the diversity of subjects, and usually analyze based on experimental data of six to 20 subjects in the experimental validation. Some methods provide the gender and age of the subjects (Ma et al., 2014; Mestre et al., 2018; Wang and Ji, 2018; Uhm et al., 2020; Chen and Shi, 2023). Whether glasses are allowed to be worn is also an issue that should be considered, as wearing glasses not only blocks the eyes, but also causes reflection from the glasses. Some methods explicitly state that wearing glasses is not allowed (Lai et al., 2015; Wang and Ji, 2016; Xia et al., 2016; Liu J. et al., 2021). Eom et al. (2019) studied the gaze estimation method when wearing glasses, and learned a neural network from the center of black eye, inner and outer corners, and gaze direction using the samples collected when the user looked at nine calibration points on the screen. To reduce the impact of individual differences and enable the model to perform well on more individuals, the subject situation should be fully considered.

### 3.3 Experimental distance

The experimental distance is usually determined by the imaging range of the camera. To enable the required features to be imaged and ensure the imaging quality, there are specific requirements for the experimental distance. In most personal calibration processes, the distance between the user and the screen is within the range of 300–800 mm (Blignaut, 2013; Ma et al., 2014; Chen and Ji, 2015; Lai et al., 2015; Shin et al., 2015; Sun et al., 2015; Li and Li, 2016; Wang and Ji, 2016, 2018; Cheng et al., 2017; Hu et al., 2019; O'Reilly et al., 2019; Uhm et al., 2020; Liu J. et al., 2021), which is consistent with the scenario of using a computer. Some personal calibration processes are conducted in scenarios larger than 1 m, such as experiments in head-mounted systems (Mansouryar et al., 2016; Liu M. et al., 2020), or in vehicle driving scenarios (Yuan et al., 2022). Mansouryar et al. (2016) set the experimental distance to 1 m/1.25 m/1.5 m/1.75 m/2 m to investigate whether calibration at a single depth is sufficient, and concluded that the estimation performance can be improved with multiple calibration depths.

### 3.4 Calibration benchmark

The calibration benchmark here refers to the reference information used for calibration. In most cases, the set calibration benchmark is several on-screen calibration points with known coordinates (Blignaut, 2013; Ma et al., 2014; Lai et al., 2015; Shin et al., 2015; Sun et al., 2015; Wang and Ji, 2016; Xia et al., 2016; Cheng et al., 2017; Mestre et al., 2018; Hu et al., 2019; O'Reilly et al., 2019; Uhm et al., 2020; Liu J. et al., 2021). Blignaut (2013) compared the gaze estimation performance using a mapping model from pupil-glint vector to 2D POR when the number of calibration points was 5/9/14/18/23/135. He found that the gaze accuracy can reach 0.5° when the number of calibration points was not < 14. In contrast, Li and Li (2016) used an RGB camera as a calibration benchmark, and asked subjects to gaze at the RGB camera from different head positions to calibrate the eyeball center in the head coordinate system. Wang and Ji (2018) utilized complementary gaze constraint, center prior constraint, display boundary constraint, and angular constraint to obtain the kappa angle of left and right eyes through implicit personal calibration, without the need for known fixed calibration points. There are also some methods that can obtain saliency maps as the calibration benchmark for subjects to view images (Chen and Ji, 2015; Yan et al., 2017; Liu M. et al., 2020).

### 3.5 Head movement

In addition to being constrained by the camera's imaging range, the setting of head movement is mainly determined by the gaze tracking algorithm. For example, when constructing a mapping model from pupil/iris-corner vector or pupil/iris-glint vector to 2D POR using personal samples, the gaze accuracy would decrease significantly with head movement (Morimoto and Mimica, 2005; Sigut and Sidha, 2011). In 3D geometry-based methods, head rolling will generate a rotational component of the eye's OA around itself, which cannot be characterized by the eye visual features or the centers located on the OA. Even using a system with light sources, the glint formed by corneal reflection of the light source cannot reflect the rotational component due to the approximate spherical structure of the cornea. Therefore, some 3D gaze estimation methods limit head rotation (Li and Li, 2016; Wang and Ji, 2018; Liu J. et al., 2021). Reasonable setting of head movement can ensure the accuracy of personal calibration and provide conditions for accurate gaze estimation.

## 4 Personal calibration modes

Personal calibration plays an important role in gaze tracking as it can obtain user-specific parameters and improve gaze estimation performance. However, complex personal calibration not only increases the burden on users, but also limits its application range. This section discusses four personal calibration modes based on their complexity: explicit multi-point calibration, explicit single-point calibration, implicit/automatic calibration, and calibration-free.

### 4.1 Explicit multi-point calibration

Explicit multi-point calibration is the most common calibration mode. 2D mapping-based methods usually conduct an explicit calibration process where users stare at multiple calibration points to obtain sufficient data to calibrate the required personal information, thereby determining the gaze estimation model (Blignaut, 2013; Ma et al., 2014; Cheung and Peng, 2015; Shin et al., 2015; Jen et al., 2016; Mansouryar et al., 2016; Xia et al., 2016; Arar et al., 2017; Cheng et al., 2017; Mestre et al., 2018; Sasaki et al., 2018, 2019; Hu et al., 2019; Luo et al., 2020; Morimoto et al., 2020; Uhm et al., 2020). Nine calibration points are the most commonly used. Cheung and Peng (2015) used nine calibration points to determine the mapping model from iris-corner vector to POR. Subsequently, the AWPOSIT algorithm was used to compensate for the displacement of head movement based on the static POR. During personal calibration, the subjects were allowed to wear glasses. Luo et al. (2020) verified the proposed mapping equation based on homography transformation by using nine calibration points in a single-camera-single-light-source system with a head entrust stent. Compared with the calibrated classical quadratic polynomial mapping equation (with an accuracy of 0.99°), the gaze accuracy was within 0.5°. Although simplifying the mapping model can reduce the number of calibration points, at least two calibration points are required (Xia et al., 2016; Morimoto et al., 2020). For 3D geometry-based methods, if a single-camera system or depth sensor is used, at least five calibration points are usually used to calibrate the required eye parameters (Xiong et al., 2014; Wang and Ji, 2016; Brousseau et al., 2018; O'Reilly et al., 2019; Liu J. et al., 2020). Wang and Ji (2016) estimated the kappa angle, eyeball radius, distance between 3D corneal center and 3D eyeball center, as well as 3D eyeball center in the head coordinate system using five calibration points. Liu J. et al. (2020) determined the iris radius and kappa angle of subjects through five-point calibration, and the head roll was not allowed during calibration. In contrast, appearance-based methods typically do not require an explicit multi-point calibration process to obtain calibration samples.

### 4.2 Explicit single-point calibration

To avoid staring at multiple calibration points, researchers attempted to improve the gaze tracking algorithm by using a single calibration point to obtain personal information. The implementation of explicit single-point calibration for 2D mapping-based methods usually requires the use of some prior knowledge. Choi et al. (2014) constructed a user calibration database based on multi-point calibration in advance, which can then achieve gaze tracking by looking at a single calibration point. Yoon et al. (2019) combined the prior knowledge of average calibration with single-point calibration when determining the mapping model. Most explicit single-point calibration processes are conducted in 3D geometry-based methods. Using a single-camera-multi-light-source system or multi-camera system, CC-based method can achieve 3D gaze estimation with free head movement through single-point calibration by fully utilizing the geometric imaging model (Guestrin and Eizenman, 2007; Nagamatsu et al., 2008, 2021; Villanueva and Cabeza, 2008; Ebisawa and Fukumoto, 2013; Lai et al., 2015). DS-based methods can also calibrate the eyeball center and kappa angle in the head coordinate system through explicit single-point calibration. However, unlike the above calibration process, users need to focus on this calibration point at multiple head positions. Zhou et al. (2017) pointed out that each subject required to look at a given calibration point with two different head poses during calibration, while they asked the subject to gaze at the calibration point with 10 different head poses or gaze directions to obtain a more accurate estimation. For appearance-based methods, explicit single-point calibration is used to obtain several calibration images for gaze compensation (Liu G. et al., 2021; Chen and Shi, 2023).

### 4.3 Implicit/automatic calibration

Explicit calibration requires users to stare at known calibration points, and multi-point calibration is time consuming. Although the number of calibration points can be reduced to one, multi-point calibration shows stronger robustness than single-point calibration (Guestrin and Eizenman, 2007; Morimoto et al., 2020), and the naturalness of interaction is more pursued by people. Therefore, studying implicit/automatic calibration methods has become a major research trend.

On one hand, to avoid the need for known calibration points, the natural constraint that the gazes of left and right eyes converge at one point is often used (Model and Eizenman, 2010; Wang and Ji, 2018; Wen et al., 2020). Model and Eizenman (2010) automatically calibrated the kappa angle in a two-camera-four-light-source system by minimizing the distance between the intersections of the VAs of left and right eyes with one or more observation surfaces. Wen et al. (2020) proposed a personal calibration process that requires users to focus on a specific yet unknown point and move their heads (rotate and translate) while maintaining focus. Based on the constraint of minimizing the distance between the PORs of left and right eyes, the parameters including eyeball radius, 3D eyeball center, and kappa angle were calibrated.

On the other hand, utilizing visual saliency to obtain gaze information is also an effective way (Chen and Ji, 2015; Wang et al., 2016; Alnajar et al., 2017; Hiroe et al., 2018; Liu M. et al., 2020). Hiroe et al. (2018) proposed an implicit personal calibration method using the saliency map around the OA of the eye, where the peak of the mean saliency map was used to represent the VA in the eyeball coordinate system. Liu M. et al. (2020) conducted a calibration process where users can randomly scan the surrounding environment, and then established a mapping model using gaze vectors and possible 3D calibration targets in the scene calculated using saliency maps. Especially, Sugano et al. (2015) used a monocular camera to capture the user's head pose and eye images during mouse clicks, and incrementally updated the local reconstruction-based gaze estimation model by clustering the head poses, to learn the mapping between eye appearance and gaze direction continuously and adaptively. Yuan et al. (2022) automatically calibrated the gaze estimation model through gaze pattern learning in driving scenarios, where the representative time samples of forward-view gaze zone, left-side mirror, right-side mirror, and rear-view mirror, speedometer, and center stack were implicitly selected as calibration points. Implicit/automatic calibration methods can gradually adapt the system to users and improve the performance during interaction (Sun et al., 2014; Chen and Ji, 2015).

### 4.4 Calibration-free

Most calibration-free methods are based on facial or eye appearance, and researchers directly use existing datasets to train user-independent gaze estimation models (Wood et al., 2015, 2016; Wen et al., 2017a,b; Li and Busso, 2018; Cheng et al., 2020a,b, 2021; Liu S. et al., 2020; Zhang et al., 2020, 2022; Bao et al., 2021; Cai et al., 2021; Murthy and Biswas, 2021; Abdelrahman et al., 2022; Donuk et al., 2022; Hu et al., 2022; Zhao et al., 2022; Huang et al., 2023; Ren et al., 2023). Hu et al. (2022) trained the gaze estimation model using saliency features and semantic features from the DR(eye)VE dataset. Liu S. et al. (2020) took a full-face image as input, and estimated the 3D gaze by using a multi-scale channel unit and a spatial attention unit for selecting and increasing important features, respectively. Appearance-based methods mainly use the 3D eyeball center to estimate the gaze, however, the VA of the eyeball passes through the corneal center, not the eyeball center, so this is an approximate estimation. For 2D mapping-based methods and 3D geometry-based methods, the implementation of calibration-free mainly relies on parameter settings or model approximation. The former uses the parameters from classical eyeball model or population averages to set some parameters that originally need to be calibrated (Morimoto et al., 2002; Coutinho and Morimoto, 2006, 2013), such as setting the scale factor to 2 in the CR-based method (Coutinho and Morimoto, 2006); setting the corneal radius to 7.8 mm (Coutinho and Morimoto, 2013). The latter does not consider the kappa angle and directly approximates the OA of the eyeball to represent the VA of the eyeball (Shih et al., 2000). To reduce the error caused by this approximation, researchers propose the binocular model and estimate the POR using the midpoint of the POAs of left and right eyes (Hennessey and Lawrence, 2009; Nagamatsu et al., 2010, 2011). Nagamatsu et al. (2011) used four cameras and three light sources to calculate the corneal center and the OA direction based on a geometric imaging model, thereby determining the OA of a single eye. They used the midpoint of the POAs of left and right eyes as a good approximation of the POR.

### 4.5 Discussion

**Figure 5:**
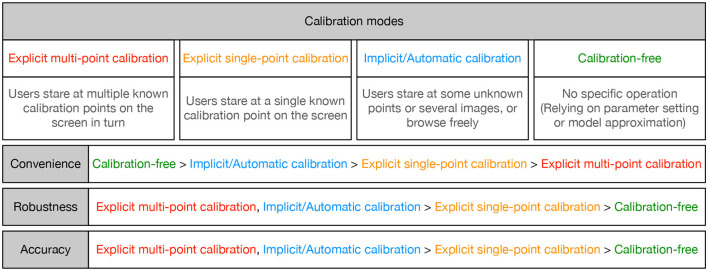
Characteristics of different calibration modes.

The characteristics of different personal calibration modes are shown in Figure 5. To obtain personal information, explicit multi-point calibration requires users to focus on multiple known calibration points on the screen in sequence, while explicit single-point calibration requires users to focus on a known calibration point. Implicit/automatic calibration collects personal information of users while looking at unknown points or images, or browsing freely. The calibration-free mode does not require the user to perform any specific operations, it is mainly implemented based on parameter settings or model approximation. From the perspective of convenience and user experience, calibration-free is the best mode, followed by implicit/automatic calibration, while explicit multi-point calibration leads to a heavier burden on the user. From the perspective of robustness and accuracy, explicit multi-point calibration and implicit/automatic calibration have stronger robustness and higher accuracy due to the use of more personal information, which is superior to explicit single-point calibration and calibration-free. Nagamatsu et al. (2008) pointed out that the accuracy of a calibration-free gaze tracking system is generally lower than that of a tracking system with a calibration, and there is a trade-off between accuracy and calibration mode. Each personal calibration mode has its own characteristics, and the selection of calibration mode should be based on practical application requirements. For example, virtual reality helmets currently use explicit multi-point calibration to ensure the high accuracy and robustness of the device. Taking the Pico G2 4K virtual reality all-in-one headset as an example, the calibration mode used is for users to gaze at five calibration points that appear sequentially on the virtual screen.

Through the analysis of different personal calibration modes, it can be seen that the personal calibration modes used in different gaze tracking methods have certain regularity. Table 2 lists the personal calibration modes in some gaze tracking methods. 2D mapping-based methods mainly adopt explicit multi-point calibration. Explicit single-point calibration can be achieved by simplifying the mapping model, but prior knowledge is required. 3D geometry-based methods can simplify the explicit calibration process by using a more complex system or improving the gaze tracking algorithm. They can also utilize natural constraints such as “the gazes of left and right eyes converge at one point” for implicit/automatic calibration. To avoid personal calibration, some user-specific parameters can be set using the parameters from classical eyeball model or population averages, or the OA of the eyeball can be approximated to represent the VA of the eyeball, thus avoiding kappa angle calibration in some single-point calibration methods. With the continuous increase of datasets, there has been a significant increase in calibration-free gaze tracking methods proposed based on appearance in recent years. However, due to individual differences, head pose differences, and environmental differences, etc., the generalization ability of appearance-based gaze estimation models is far from reaching the level of universal applications. Table 3 lists the accuracy of some state-of-the-art methods. It can be seen that under different system configurations and calibration modes, the gaze accuracy of 2D mapping-based methods and 3D geometry-based methods is generally within 2°, while the gaze accuracy of appearance-based methods is usually above 3°, even if calibration samples are used.

**Table 2:** Personal calibration modes in some gaze tracking methods.

**Calibration modes**	**2D mapping-based methods**	**3D geometry-based methods**	**Appearance-based methods**
Explicit multi-point calibration	Hu et al., 2019, Mestre et al., 2018, Cheng et al., 2017, Uhm et al., 2020, Xia et al., 2016, Blignaut, 2013, Ma et al., 2014, Shin et al., 2015, Cheung and Peng, 2015, Eom et al., 2019, Jen et al., 2016, Arar et al., 2017, Arar et al., 2015, Morimoto et al., 2020, Sasaki et al., 2018, Sasaki et al., 2019, Luo et al., 2020, Coutinho and Morimoto, 2006	Wang and Ji, 2016, Li and Li, 2016, Liu J. et al., 2021, O'Reilly et al., 2019, Xiong et al., 2014, Mansouryar et al., 2016, Brousseau et al., 2018, Liu J. et al., 2020, Ebisawa and Fukumoto, 2013	Liu G. et al., 2021, Chen and Shi, 2023, Wang et al., 2023
Explicit single-point calibration	Choi et al., 2014, Yoon et al., 2019	Lai et al., 2015, Zhou et al., 2017, Sun et al., 2015, Zhou et al., 2016, Villanueva and Cabeza, 2008, Guestrin and Eizenman, 2007, Nagamatsu et al., 2008, Ebisawa and Fukumoto, 2013	
Implicit/automatic calibration	–	Wang et al., 2016, Wang and Ji, 2018, Liu M. et al., 2020, Sun et al., 2014, Ebisawa and Fukumoto, 2013, Wen et al., 2020, Model and Eizenman, 2010	Alnajar et al., 2017, Yuan et al., 2022, Sugano et al., 2015, Bao et al., 2021, Wu et al., 2022
Calibration-free	Yan et al., 2017, Hennessey and Lawrence, 2009	Morimoto et al., 2002, Shih et al., 2000, Hennessey and Lawrence, 2009, Nagamatsu et al., 2010, Nagamatsu et al., 2011	Hu et al., 2022, Li and Busso, 2018, Zhang et al., 2020, Cheng et al., 2020b, Liu S. et al., 2020, Cheng et al., 2020a, Murthy and Biswas, 2021, Cheng et al., 2021, Cai et al., 2021, Zhang et al., 2022, Zhao et al., 2022, Abdelrahman et al., 2022, Wood et al., 2015, Wood et al., 2016, Wen et al., 2017b, Wen et al., 2017a, Donuk et al., 2022, Ren et al., 2023

**Table 3:** Comparison of the state-of-the-art methods using different personal calibration modes.

**References**	**Methods**	**Systems/datasets**	**Calibration modes**	**Accuracy**
Hu et al. (2019)	2D mapping-based	Single camera	Explicit multi-point calibration	2.67°
Cheng et al. (2017)	2D mapping-based	Single camera, five IR LEDs	Explicit multi-point calibration	0.7°
Uhm et al. (2020)	2D mapping-based	Single camera, four IR light sources	Explicit multi-point calibration	0.80°± 0.72°
Arar et al. (2017)	2D mapping-based	Single camera, five groups of NIR LEDs	Explicit multi-point calibration	1.0°
Sasaki et al. (2019)	2D mapping-based	A polarization camera	Explicit multi-point calibration	1.46°
Yoon et al. (2019)	2D mapping-based	Driving scenario	Explicit single-point calibration	79.28%
Yan et al. (2017)	2D mapping-based	Single camera, four IR light sources	Calibration-free	47 mm
Wang and Ji (2016)	3D geometry-based	A depth sensor(Kinect)	Explicit multi-point calibration	4.0°
Liu J. et al. (2021)	3D geometry-based	Single camera, single light source	Explicit multi-point calibration	X:1.15°, Y:1.1°
O'Reilly et al. (2019)	3D geometry-based	Single camera, nine LEDs	Explicit multi-point calibration	0.69°
Zhou et al. (2017)	3D geometry-based	A depth sensor (Kinect)	Explicit single-point calibration	1.99°
Wang and Ji (2018)	3D geometry-based	Single camera, four IR light arrays	Implicit/automatic calibration	1.3°
Wen et al. (2020)	3D geometry-based	A web camera	Implicit/automatic calibration	3.45°
Liu G. et al. (2021)	Appearance-based	EYEDIAP, MPIIGaze, UT-Multiview	Explicit calibration	2.99°–3.88°
Chen and Shi (2023)	Appearance-based	MPIIGaze, EYEDIAP, ColumbiaGaze, NISLGaze	Explicit calibration	2.6°–3.5°
Wu et al. (2022)	Appearance-based	GazeCapture, MPIIGaze, EYEDIAP, Gaze360	Implicit/automatic calibration	4.37°
Bao et al. (2021)	Appearance-based	GazeCapture, MPIIFaceGaze	Implicit/automatic calibration	4.4°
Zhao et al. (2022)	Appearance-based	GazeCapture, MPIIGaze, ETH-XGaze	Calibration-free	4.47°
Murthy and Biswas (2021)	Appearance-based	MPIIGaze, RT-Gene	Calibration-free	4.09°

## 5 Performance simulations of typical personal calibration methods

To indicate the characteristics of different settings in personal calibration intuitively, this section conducted some comparative simulation experiments on typical personal calibration methods for 2D and 3D gaze estimation, to reflect several key issues of personal calibration and provide reference for researchers to design personal calibration.

### 5.1 Personal calibration simulations in PCRT/ICRT-based methods

The PCRT/ICRT-based method is the most commonly used method for 2D gaze estimation, and is the core eye tracking technology used by many eye tracking device manufacturers. It essentially establishes a mapping model from pupil/iris-glint vector to 2D POR through personal calibration, and uses the calibrated mapping model for 2D gaze estimation and tracking. The mapping effect of the calibrated model determines the gaze estimation performance. There are two main factors that affect the mapping effect: the 2D mapping model used and the number of calibration points used for mapping model calibration. Therefore, we discussed the performance of using different 2D mapping models and using different numbers of calibration points in personal calibration, even the distribution of calibration points.

#### 5.1.1 Comparison of 2D mapping models

**Figure 6:**
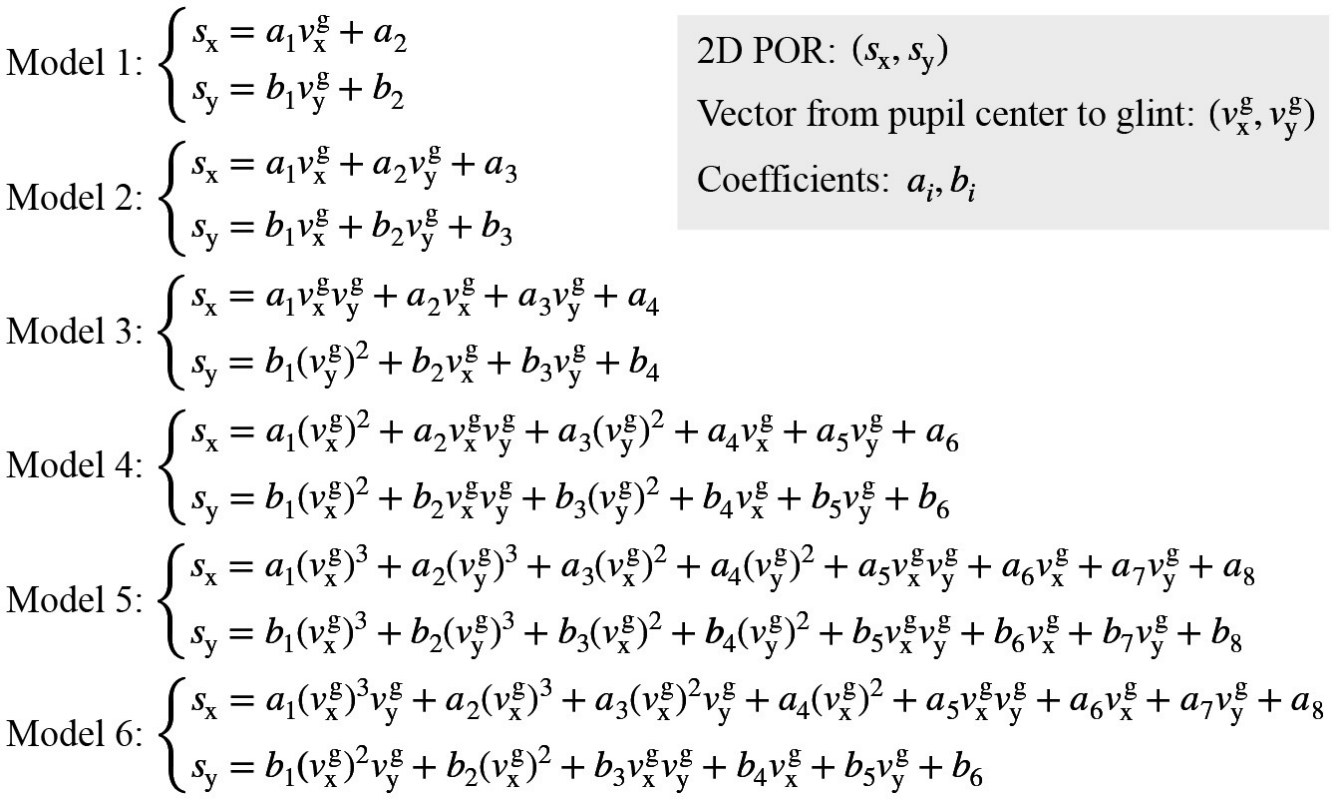
Six mapping models for analysis.

Various mapping models can be selected, such as linear, quadratic, or higher-order. Some researchers have specifically studied different 2D mapping models and stated that higher order polynomials do not noticeably improve system behavior (Cerrolaza et al., 2008; Blignaut, 2013), so we focused on performance analysis of polynomials below fourth order, and selected six commonly used mapping models from existing literature that cover different orders and forms, as shown in Figure 6. In the models, *s*_x_, *s*_y_ are the horizontal and vertical coordinates of the 2D POR, $v_{\text{x}}^{\text{g}}$, $v_{\text{y}}^{\text{g}}$ are the horizontal and vertical components of the corresponding vector from pupil center to glint, and *a*_*i*_, *b*_*i*_ are the coefficients of mapping models.

**Figure 7:**
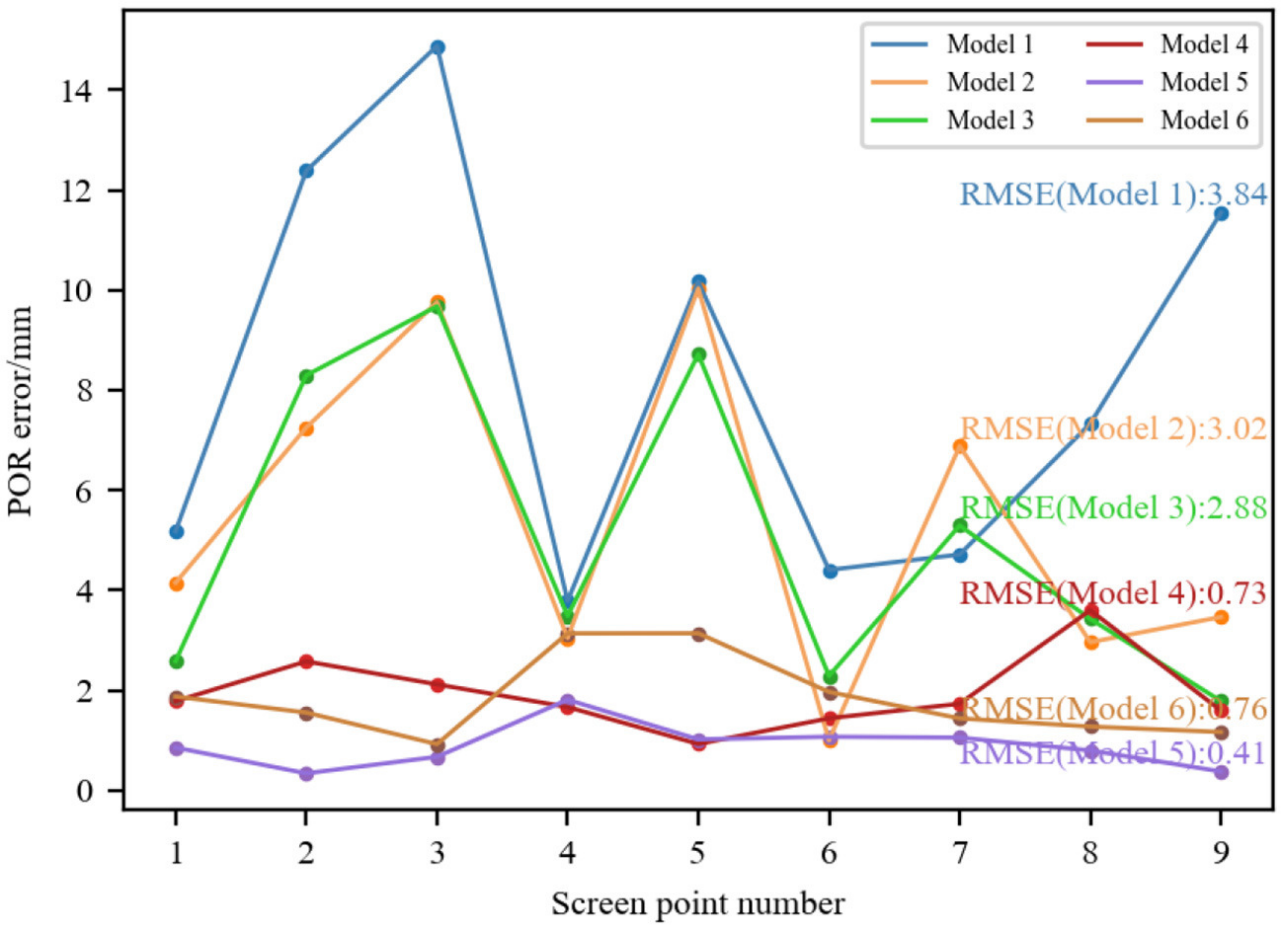
Performance comparison of calibrating different 2D mapping models.

To compare these six models, we set simulation parameters based on the eyeball structure and the geometric imaging model. The simulation parameters are as follows: the light source was set at (–120, –3, 10), the camera focal length was 6 mm, and the corneal radius was 7.8 mm. The 3D corneal center was set at (–42.7208, –60.4287, 332.4721), and the coordinates of glint were (0.7968, 1.0908, –6). Nine evenly distributed screen points were located at: (100, 75), (200, 75), (300, 75), (100, 150), (200, 150), (300, 150), (100, 225), (200, 225), (300, 225), and the coordinates of their corresponding pupil imaging centers were (0.801, 1.0201, –6), (0.7718, 1.0185, –6), (0.745, 1.0233, –6), (0.8031, 1.0375, –6), (0.7739, 1.0358, –6), (0.7471, 1.0392, –6), (0.8036, 1.0568, –6), (0.7765, 1.0554, –6), (0.75, 1.0567, –6). Using the coordinates of pupil imaging centers and glint, the vector from pupil center to glint corresponding to each screen calibration point was calculated. By taking the same nine sets of pupil-glint vector and the screen calibration point as input, the coefficients (*a*_*i*_, *b*_*i*_) in the six different models can be fitted to determine the mapping models. To analyze the performance of determined mapping models, the pupil-glint vector corresponding to each screen point was substituted into each mapping model to predict the 2D POR. The Euclidean distance between the predicted POR and the ground-truth of screen point was calculated to represent the POR error, as Figure 7 shows. When using different 2D mapping models, the root mean squared errors (RMSEs) of the nine PORs were also calculated. It can be seen that increasing the model order can reduce the POR error to a certain extent, but more complex mapping model does not have better gaze estimation performance absolutely. On the contrary, more complex models have more coefficients to be calibrated, which requires more calibration points and increases the complexity of personal calibration. Therefore, on the basis of meeting certain performance requirements, it is superior to select a simpler model. At present, the most widely used is Model 4.

#### 5.1.2 Comparison of the number of calibration points

The selection of the number of calibration points should consider the minimum number required for the mapping model. For Model 4, there are six coefficients in each polynomial that need to be determined through personal calibration, so the number of calibration points used for this model is generally not less than six. To improve the performance, more calibration points may be used. Here, we analyzed the impact of the number of calibration points used in personal calibration on its performance.

**Figure 8:**
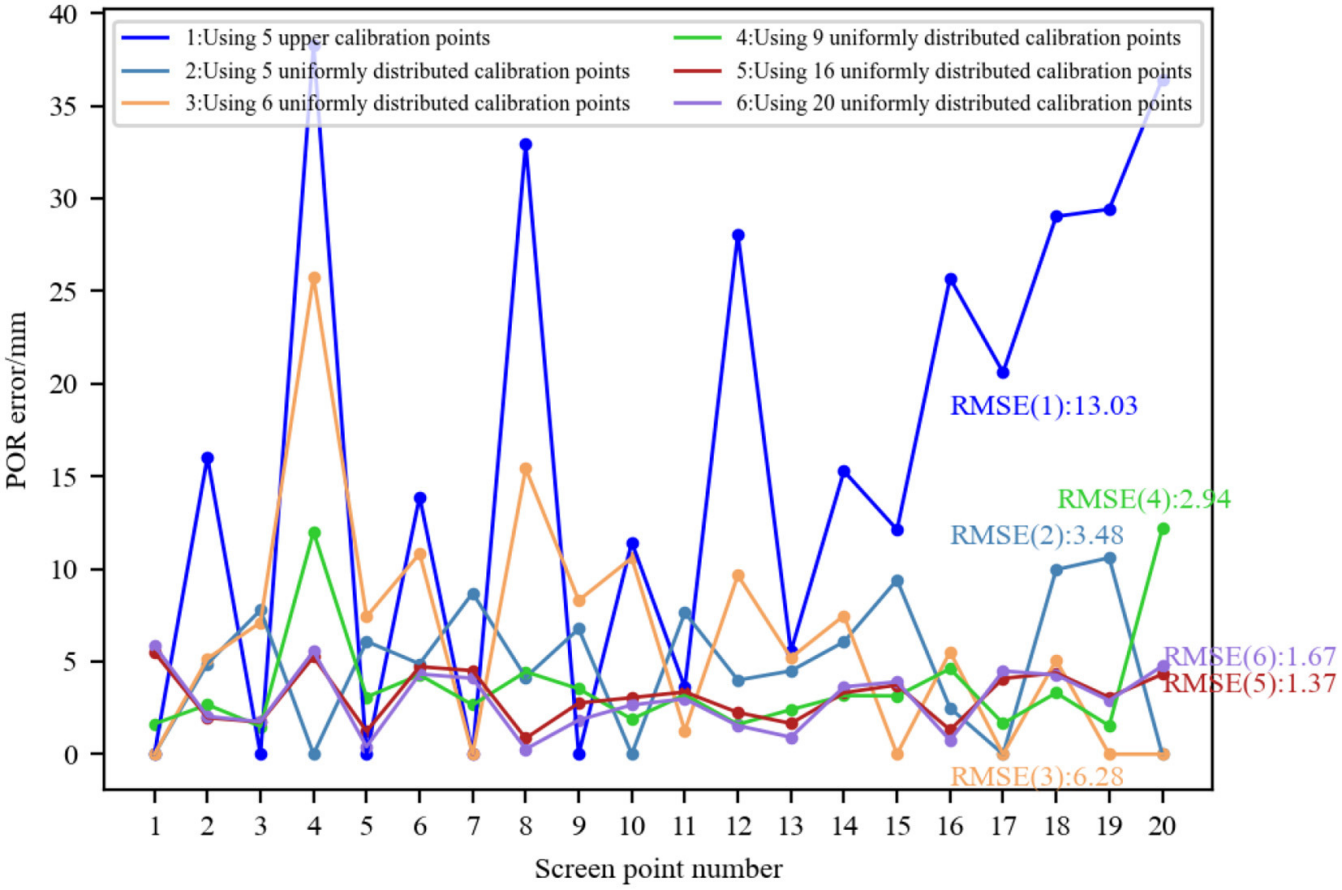
Performance comparison of 2D mapping models calibrated with different numbers of calibration points.

The simulation parameters are set as follows: the light source, camera focal length, and corneal radius were the same as before. Twenty evenly distributed screen points (five rows and four columns) were set. Under the constraint that the distance between the 3D corneal center and the screen is 300 mm, the 3D corneal center was randomly generated, and the glint was determined accordingly by the corneal reflection of light source. Then the corresponding pupil imaging center for each screen point was determined based on the eyeball structure and the pupil refraction. Since Model 4 is the most widely used model, here, we used it to compare the gaze estimation performance when the number of calibration points is 5/6/9/16/20, respectively. Twenty evenly distributed screen points were numbered clockwise in a row. When studying 20 calibration points, the pupil-glint vectors and screen point coordinates corresponding to all screen points were used to calibrate the mapping model. When studying 5/6/9/16 calibration points, the calibration data was selected from the data of 20 screen points to ensure a single variable. Considering the generalization ability of the mapping model, the scattered calibration points on the screen were used as much as possible in our simulation. When studying five upper calibration points, the selected screen points were [1, 3, 5, 7, 9]; When studying five uniformly distributed calibration points, the selected screen points were [1, 4, 10, 17, 20]. When studying six calibration points, the selected screen points were [1, 7, 15, 17, 19, 20]. When studying nine calibration points, the selected screen points were [1–3, 10–12, 17–19]. When studying 16 calibration points, the selected screen points were [1–8, 13–20]. Using the pupil-glint vectors and screen point coordinates corresponding to these screen points as calibration data, the coefficients of Model 4 were fitted using different amounts of calibration data to determine the 2D mapping model. Then the 2D POR was predicted by substituting the pupil-glint vector corresponding to each screen point into the calibrated mapping model. The Euclidean distance between the predicted 2D POR and the ground-truth of screen point was calculated to represent the POR error, and the RMSEs of the 20 PORs when the mapping model was calibrated using different numbers of calibration points were also calculated, as shown in Figure 8. When using five calibration points, if all the points are in the upper part of the screen, the POR error was relatively large. In contrast, if the calibration points are uniformly distributed, the POR error was relatively small. When using six calibration points, small POR errors only exist at the screen points used as calibration points. This is because fewer calibration points cannot accurately represent the mapping relationship between eye features and any screen position. When there are less than six calibration points, polynomial fitting is underdetermined; When using six calibration points, there may be significant errors at certain points due to the inconsistency between the mapping relationship and the fitted mapping model. In contrast, when using more than six calibration points, the calibrated mapping model achieved high gaze accuracy. Therefore, the gaze estimation performance can be improved by increasing the number of calibration points, but it comes at the cost of increasing the complexity of personal calibration.

### 5.2 Personal calibration simulations in CC-based methods

Most 3D gaze estimation methods are based on common cameras, which estimate eye parameters such as corneal center and pupil center according to the basic structure and the geometric imaging model of the eyeball, in order to construct the OA, and then use the kappa angle to convert the 3D gaze. In this process, to take individual differences into consideration, user-specific parameters such as corneal radius and kappa angle are usually obtained through a personal calibration process. Therefore, based on our experience, we explored the two most prominent issues in the personal calibration for 3D gaze estimation. One is the setting of calibration parameters when calibrating the user-specific corneal radius, and the other is the selection of calibration modes when calibrating the kappa angle.

#### 5.2.1 Comparison of calibration parameters

When using the CC-based method in a single-camera system, it is usually necessary to calibrate the user-specific corneal radius. Here, the importance of selecting calibration parameters is emphasized by analyzing several calibration parameter settings when calibrating the corneal radius. To replicate the typical CC-based method (Guestrin and Eizenman, 2006), the simulation parameters are set as follows: we set two light sources, located at (–120, –3, 10) and (120, –3, 10). The camera focal length was 6 mm. The ground-truth of corneal radius was 7.8 mm, and the distance between the 3D corneal center and the 3D pupil center was 4.2 mm. The horizontal and vertical components of kappa angle were 5 and 1.5°, respectively. We set nine evenly distributed screen points, and randomly generated the 3D corneal center corresponding to each screen point based on the constraint of 500 mm distance from the 3D corneal center to the screen. Using the 3D corneal center and corneal radius, the glints formed by two light sources were calculated based on the corneal reflection.

During corneal radius calibration, the parameters used include the positions of two light sources and their corresponding glints. We used the same nine sets of data (corresponding to nine evenly distributed screen points) for testing, with only the set calibration parameters being different. According to the corneal reflection of the light source, there are: ① the distance between the reflection point and the 3D corneal center is equal to the corneal radius; ② the incident light, normal, and reflected light are coplanar; ③ the incident angle is equal to the reflection angle. Using these properties, we studied three calibration parameter settings: (1) the calibration parameters were directly corneal radius *R* and 3D corneal center ***C***. According to ①, the reflection points were represented by *R* and ***C***. Then, four equations were constructed using ② and ③ to optimize the solutions of *R* and ***C***; (2) According to ②, the reflection planes of two light sources contain the 3D corneal center ***C*** and the camera optical center ***O***, so the vector ***OC*** was obtained by intersecting the reflection planes of two light sources. Then, the 3D corneal center ***C*** was represented by the product of a proportional coefficient *t* and the vector ***OC***. The reflection point ***G***_*i*_(*i* = 1, 2) was represented by the product of the proportional coefficients *u*_*i*_ and the corresponding glint ***g***_*i*_. Four unknowns were *R*, *t*, *u*_*i*_, and they were optimized by constructing four equations using ① and ③; (3) On the basis of (2), *u*_*i*_ was represented by using *R* and ***C*** according to ①, thereby the reflection point ***G***_*i*_ was represented by *u*_*i*_ and ***g***_*i*_. At this time, there were only two unknowns: *R* and *t*. Two equations were constructed using ③ for optimization.

We used the least square method to solve the above calibration parameters. To test the robustness of the calibration results, we set three different initial values for each calibration parameter setting. The corneal radius error obtained through calibration is shown in Table 4. The results of directly calibrating *R* and ***C*** depend on the setting of initial values, and it is not easy to set the initial coordinates of 3D corneal center. The latter two calibration parameter settings first use the intersection of two corneal reflection planes to determine the vector ***OC***, ensuring the absoluteness of the 3D corneal center ***C*** on the line OC and eliminating the deviation of 3D corneal center ***C***. Within a certain range of initial values, the corneal radius can be accurately calibrated. However, it is superior to reduce the calibration parameters to *R* and *t* to avoid the initial setting of some non-intuitive parameters (e.g., *u*_*i*_ needs to be determined by roughly estimating the ratio of the reflection point ***G***_*i*_ to the glint ***g***_*i*_).

**Table 4:** Comparison of corneal radius calibration under different calibration parameter settings.

**Calibration parameters**	**Initial values**	**Calibrated corneal radius errors of different calibration points/mm**								
		**1**	**2**	**3**	**4**	**5**	**6**	**7**	**8**	**9**
*R*, ***C***	[7, –30, –80, 500]	–0.8	–0.8	–0.8	–0.8	–0.8	–0.8	–0.8	–0.8	–0.8
	[8, –20, –60, 400]	0.2	0.2	0.2	0.2	0.2	5.7 × 10^−4^	–1.6 × 10^−5^	0.2	5.4 × 10^−4^
	[7.8, –30, –80, 500]	0	0	0	0	0	0	0	0	0
*R*, *t*, *u*_*i*_	[7, 400, –60, –60]	2.3 × 10^−4^	-1.3 × 10^−6^	8.3 × 10^−6^	5.9 × 10^−6^	–1.4 × 10^−5^	–1.6 × 10^−5^	2.3 × 10^−5^	3.7 × 10^−6^	1.0 × 10^−5^
	[8, 400, –60, –60]	2.4 × 10^−4^	5.6 × 10^−6^	1.0 × 10^−5^	1.2 × 10^−5^	1.1 × 10^−5^	5.7 × 10^−4^	–1.6 × 10^−5^	2.2 × 10^−5^	5.4 × 10^−4^
	[8, 500, –80, –80]	4.0 × 10^−6^	1.1 × 10^−7^	–1.4 × 10^−2^	5.1 × 10^−6^	3.2 × 10^−4^	4.6 × 10^−5^	-1.2 × 10^−5^	9.9 × 10^−6^	–1.6 × 10^−5^
*R*, *t*	[7,400]	2.3 × 10^−4^	–1.3 × 10^−6^	8.3 × 10^−6^	5.9 × 10^−6^	–1.4 × 10^−5^	–1.6 × 10^−5^	2.3 × 10^−5^	3.7 × 10^−6^	1.0 × 10^−5^
	[8, 400]	2.4 × 10^−4^	5.6 × 10^−6^	1.0 × 10^−5^	1.2 × 10^−5^	1.1 × 10^−5^	5.7 × 10^−4^	–1.6 × 10^−5^	2.2 × 10^−5^	5.4 × 10^−4^
	[8, 500]	4.0 × 10^−6^	1.2 × 10^−7^	–1.4 × 10^−2^	5.1 × 10^−6^	3.2 × 10^−4^	4.6 × 10^−5^	–1.2 × 10^−5^	9.9 × 10^−6^	–1.6 × 10^−5^

#### 5.2.2 Comparison of calibration modes

Kappa angle is a significant parameter for converting from the OA to the VA of the eyeball. We compared the performance under different calibration modes by analyzing kappa angle calibration. The simulation parameters are set as follows: Using the parameters set in Section 5.2.1, the pupil imaging ellipse parameters corresponding to each screen point were calculated based on eyeball structure and pupil refraction. They are necessary in implicit multi-point calibration.

We evaluated the following five methods: (1) Explicit multi-point calibration: the OA vectors and VA vectors corresponding to nine screen points were used to calibrate a transformation matrix for converting the OA to the VA of the eyeball (Zhu and Ji, 2008; Wan et al., 2022); (2) Explicit single-point calibration: Due to the fact that the kappa angle is an eye invariant parameter, each screen point corresponds to an equal kappa angle. That is to say, any screen point can be selected as the calibration point. We selected the fifth screen point here. After calculating the horizontal and vertical angles of OA and VA in the eyeball coordinate system when staring at the fifth screen point respectively, the horizontal and vertical angles of kappa angle were calculated by subtracting the corresponding angles of OA and VA (Guestrin and Eizenman, 2006; Zhou et al., 2016); (3) Implicit multi-point calibration: the kappa angle was automatically calibrated by complementary gaze constraint, center prior constraint, display boundary constraint and angular constraint using data from nine screen points (Wang and Ji, 2018); (4) Calibration-free (set kappa): Considering that the set kappa angle deviates from the ground-truth in most cases, the horizontal and vertical components of the kappa angle were set to 5 and 1°, respectively. The difference between the vertical angle and the ground-truth was 0.5°; (5) Calibration-free (POA average): the kappa angle was not considered, and the average of the POAs of left and right eyes was used as the estimated POR (Hennessey and Lawrence, 2009; Nagamatsu et al., 2010, 2011).

**Figure 9:**
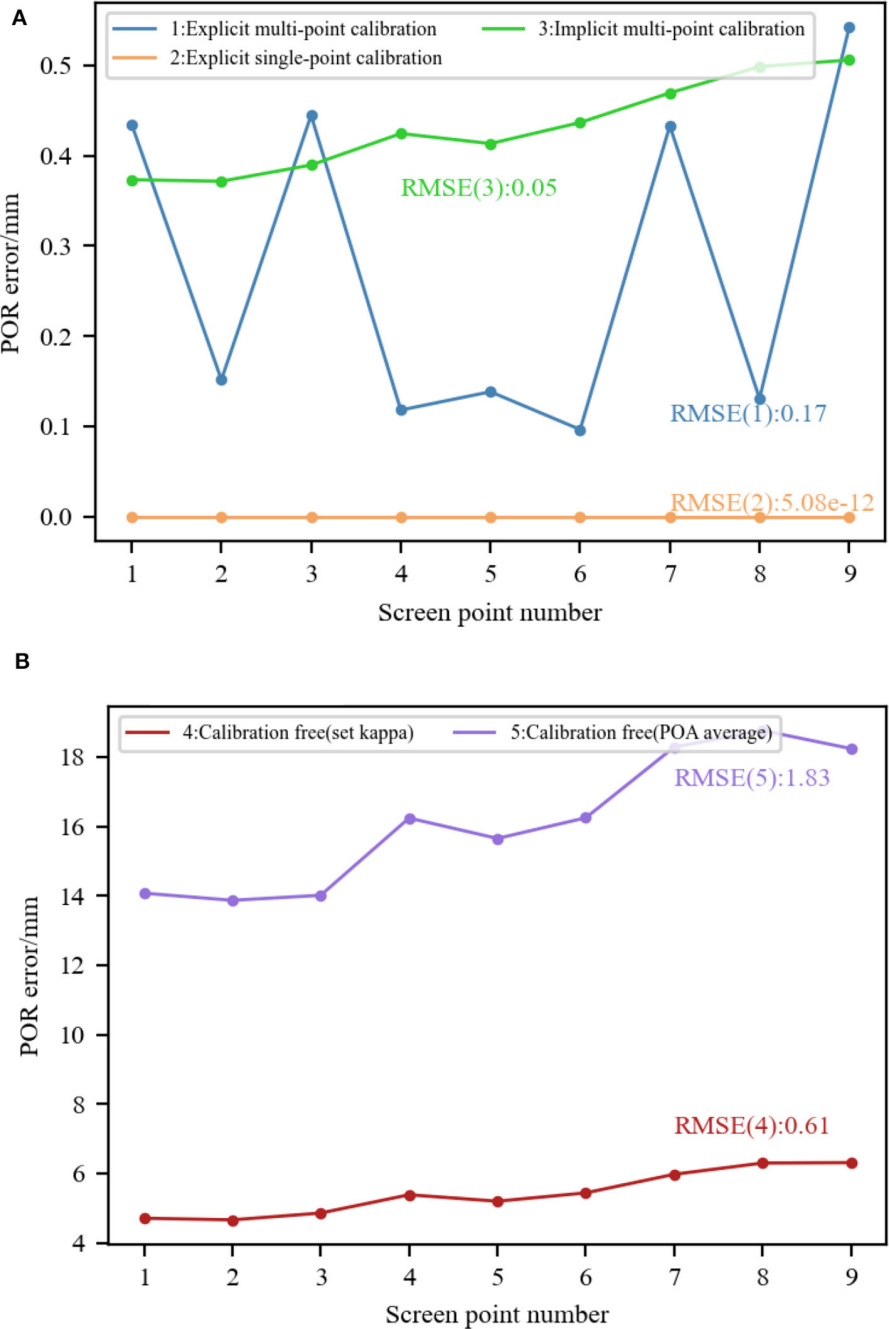
Comparison of 3D gaze estimation performance under different calibration modes. **(A)** Explicit/implicit calibration. **(B)** Calibration-free (using preset values/using the average of POAs of both eyes).

Using the same simulation data, the kappa angle calibrated using the above five methods are shown in Table 5. Then, the calibrated kappa angle parameters were used to estimate the POR when staring at each screen point. The Euclidean distance between the predicted POR and the ground-truth of screen point was calculated to represent the POR error, as Figure 9 shows. The RMSEs of the nine PORs when using different calibration modes were calculated and labeled. It can be seen that when the kappa angle obtained from explicit single-point calibration is accurate, the accurate POR can be estimated. Explicit or implicit multi-point calibration includes the optimization process, resulting in small kappa angle errors. The estimated POR error was < 1.2 mm, and the RMSE was < 0.2 mm. When using the calibration-free method with the set kappa angle, the POR error was about 5 mm due to a deviation of 0.5° in the vertical. When using the calibration-free method of averaging the POAs of left and right eyes, the POR error was about 15 mm. This is because the average operation can only offset the horizontal component of the kappa angle of left and right eyes. In our simulation model, the vertical component of the kappa angle was 1.5°, which introduces a significant error. Therefore, personal calibration is necessary in most cases.

**Table 5:** Comparison of kappa angle results under different calibration modes.

**Calibration modes**	**Calibrated/set kappa angle**
Ground-truth	H: 5.0°, V: 1.5°
Explicit multi-point calibration	$\left[\begin{array}{ccc} 1.00790465 & 0.06573575 & -0.05748697\\ -0.06765408 & 0.99772443 & -0.02423044\\ 0.05627326 & 0.02800926 & 0.99767157 \end{array}\right]$
Explicit single-point calibration	H: 5.0°, V: 1.5°
Implicit multi-point calibration	H: 5.0°, V: 1.54°
Calibration free (set kappa)	H: 5°, V: 1°
Calibration free (POA average)	–

## 6 Key issues of personal calibration

Personal calibration is a key factor affecting the performance of gaze estimation. This section discusses several key issues related to personal calibration.

### 6.1 Determination of personal calibration information

Except for appearance-based methods that can directly use the calibration images to improve the gaze estimation performance, other methods require the calculation of user-specific information through personal calibration. When determining the required personal calibration information, it is advisable to select some eye invariant parameters or parameters with specific theoretical ranges as the information that needs to be calibrated. In addition, the amount of calibration information should be minimized as much as possible. If parameters can be converted to each other, it is meaningless to set them all as the calibration information, which will also affect the accuracy of calibration results. For example, when reconstructing the eye pose in space based on the geometric imaging model, some proportional coefficients are often used. There are certain positional relationships between spatial points represented by these proportional coefficients, such as perpendicular or equidistant. In this case, these proportional coefficients can be represented by some eyeball parameters (3D corneal center and corneal radius) based on these positional relationships, to avoid error introduction when optimizing these proportional coefficients. Selecting eye invariant parameters or parameters with specific theoretical ranges as personal calibration information can also provide a basis for setting initial values during solving.

### 6.2 Settings of personal calibration process

The settings such as system configuration, experimental distance, and head movement are generally related to gaze tracking systems or gaze estimation algorithms. The experimental distance determines the imaging quality, so its variation range is relatively fixed. According to the proposed gaze tracking algorithm, there is usually a minimum standard for system configuration. For example, at least one camera and one light source are required to construct the pupil-glint vector for PCRT/ICRT-based methods. The performance can be improved by using additional system configuration. Reducing head movement can ensure the accuracy of personal calibration, but it comes at the cost of increasing user burden. For some methods that allow for free head movement or the use of a head-mounted system, head movement setting do not need to be restricted. The subject situation and calibration benchmark are relatively flexible settings. The subject situation has certain limitations in existing methods. For unconstrained gaze estimation methods, it is necessary to expand the coverage range of subjects or increase the diversity of subjects as much as possible to verify the generality of the algorithm. The setting of calibration benchmark is actually to obtain personal calibration samples. If setting more calibration benchmarks and collecting more personal samples, it is usually easier to calculate accurate personal information. But in this way, the personal calibration process that users participate in becomes more complex. Therefore, the setting of calibration benchmark needs to balance the practical requirements for performance with the user operational complexity.

### 6.3 Selection of personal calibration mode

Simplifying or exempting personal calibration has always been a research trend in gaze tracking. Although many single-point calibration, implicit/automatic calibration, and calibration-free methods have been proposed, they have only been proven theoretically feasible to a large extent. In practical applications, explicit multi-point calibration is mainly used. The current technical level is not sufficient to achieve accurate calibration-free yet universal gaze estimation. Therefore, it is necessary to conduct personal calibration. In addition, it is of great importance to consider the comfort level of user participation in personal calibration in the new situation of pursuing natural interaction. Researchers are attempting to improve the conventional explicit multi-point calibration used in gaze tracking to simpler explicit single-point calibration or more natural implicit/automatic calibration. Through the analysis of operation mode and performance, implicit/automatic calibration is a relatively optimal personal calibration mode. It obtains gaze information during the calibration process through other methods, or directly establishes connections between calibration information using some natural constraints, thereby eliminating the need for explicit calibration points. This implicit/automatic calibration mode can also update user-specific features continuously, making personal calibration information more accurate and stable.

Overall, personal calibration is a necessary means to achieve accurate gaze tracking. The operability and real-time performance of gaze tracking can be improved by calibrating the necessary personal information, setting a reasonable personal calibration process, and selecting an optimal personal calibration mode. This helps to promote gaze tracking as an important channel for natural human-computer interaction.

## 7 Conclusion

This paper provides a comprehensive overview on personal calibration for VOG-based gaze tracking. It summarizes the personal calibration information required in different gaze estimation methods, and analyzes the settings of personal calibration from five aspects: system configuration, subject situation, experimental distance, calibration benchmark, and head movement. Subsequently, several existing personal calibration modes are analyzed. The characteristics of different personal calibration settings for typical 2D and 3D methods were analyzed through simulation experiments. Finally, three key issues for designing personal calibration are discussed, namely: How to determine personal calibration information? How to set some items in personal calibration? How to select personal calibration mode? This paper has guiding significance for conducting personal calibration and promoting natural interaction.

Although many convenient personal calibration methods have been proposed in theoretical research, they are rarely implemented in industrial applications. On one hand, this is because the generality verification in theoretical research is insufficient, such as limitations on head movement and wearing glasses, which limit the application scenarios of theoretical methods. On the other hand, this is related to product requirements, and a simple personal calibration mode may not meet the performance indicators such as accuracy. To better integrate with practical application requirements, the following directions can be studied:

(1) Research on unconstrained personal calibration scenarios: Unconstrained personal calibration is a more user-friendly experience that requires minimizing the limitations in personal calibration settings to enable subjects to naturally complete the calibration process. It not only requires the calibration algorithm to be feasible in the staring state, but also requires reasonable processing of personal information collected in specific or extreme situations such as blink and saccade.
(2) Research on some general criteria or constraints in gaze patterns: implicit/automatic calibration is a relatively natural calibration mode. It is recommended to conduct refined research on some basic criteria or natural constraints and verify their universality, which can broaden the thinking of designing personal calibration and promote the application of natural calibration mode.
(3) Research on personal calibration modes in specific application scenarios: Conducting research on personal calibration modes for specific application scenarios makes it easier to balance various indicators and improve some important indicators in a targeted manner. Taking head-mounted devices as an example, the factor of head movement can be approximately ignored, while high accuracy, strong robustness and real-time performance are the indicators we pursue.

## Author contributions

JL: Conceptualization, Data curation, Funding acquisition, Investigation, Methodology, Writing – original draft, Writing – review & editing. JC: Formal analysis, Funding acquisition, Methodology, Supervision, Writing – review & editing. ZY: Conceptualization, Methodology, Validation, Writing – review & editing.
